# Trial of a Novel Intervention to Improve Multiple Food Hygiene Behaviors in Nepal

**DOI:** 10.4269/ajtmh.16-0526

**Published:** 2017-06-07

**Authors:** Om Prasad Gautam, Wolf-Peter Schmidt, Sandy Cairncross, Sue Cavill, Valerie Curtis

**Affiliations:** 1 London School of Hygiene and Tropical Medicine, London, United Kingdom; 2 Freelance Consultant, London, United Kingdom

## Abstract

In this study, we report on the results of a trial of an intervention to improve five food hygiene behaviors among mothers of young children in rural Nepal. This novel intervention targeted five behaviors; cleanliness of serving utensils, handwashing with soap before feeding, proper storage of cooked food, and thorough reheating and water treatment. Based on formative research and a creative process using the Behavior-Centered Design approach, an innovative intervention package was designed and delivered over a period of 3 months. The intervention activities included local rallies, games, rewards, storytelling, drama, competitions linking with emotional drivers of behavior, and “kitchen makeovers” to disrupt behavior settings. The effect of the package on behavior was evaluated via a cluster-randomized before–after study in four villages with four villages serving as controls. The primary outcome was the difference in the mean cluster level proportions of mothers directly observed practicing all five food hygiene behaviors. The five targeted food hygiene behaviors were rare at baseline (composite performance of all five behaviors in intervention 1% [standard deviation (SD) = 2%] and in control groups 2% [SD = 2%]). Six weeks after the intervention, the target behaviors were more common in the intervention than in the control group (43% [SD = 14%] versus 2% [SD = 2%], *P* = 0.02) during follow-up. The intervention appeared to be equally effective in improving all five behaviors in all intervention clusters. This study shows that a theory-driven, systematic approach employing emotional motivators and modifying behavior settings was capable of substantially improving multiple food hygiene behaviors in Nepal.

## Introduction

Behavior change has been described as the last-mile problem in public health.^1^ Despite the fact that solutions are available for most of the world's burden of disease, problems of uptake remain. New ways of changing behavior change at scale are needed.^2^–^4^ A case in point is food hygiene. Although poor food hygiene is implicated in morbidity and mortality globally, little attention has been paid as to how to go about changing this complex of multiple, entrenched, routine daily behaviors in domestic settings in developing countries—where interventions are most needed.

Poor food hygiene contributes to the burden of disease from diarrhea, which, despite some recent progress,^5^ still kills 700,000 under-five children every year.^6^ One estimate suggests that up to 70% of diarrheal episodes in developing countries may be caused by pathogens transmitted through food.^7^,^8^ High rates of diarrheal disease in childhood also predispose to malnutrition among young children.^9^–^11^ Diarrhea risk increases during the infant weaning period in low-income settings^12^–^15^ and child growth often falters after the initiation of weaning.^10^ Contaminated weaning foods, in particular, have been implicated in diarrheal diseases in low-income contexts,^14^,^16^ though observational studies gives inconclusive results.^17^ Weaning foods are often prepared in unhygienic conditions and infants who, until then, have consumed only breast milk, may be exposed to infective doses of food-borne pathogens.^14^,^17^ Foods also provide a route for the transmission of the agents of environmental enteropathy, which may be a cause of child malnutrition in developing countries.^18^,^19^ However, research into food hygiene has been neglected and diarrhea and undernutrition prevention programs tend to prioritize breastfeeding promotion, food and micronutrient supplementation, and immunization rather than food hygiene and safety^20^ in low-income settings.

Developing country homes provide many obstacles to safe food hygiene practice^21^,^22^ including high ambient temperatures,^17^,^23^ lack of refrigeration, poor storage facilities,^24^ inadequate sanitation and presence of animals in kitchens leading to environmental fecal contamination,^19^,^25^ lack of running water,^26^ cooking fuel scarcity,^14^ hard-to-clean household surfaces, often compounded by heavy female workloads, and poor access to information on safe hygiene. Poor practices include long gaps between meal preparation and feeding,^16^,^27^,^28^ the use of unclean utensils,^29^,^30^ the washing of utensils in contaminated water,^26^ allowing flies to access foods, not washing hands before food handling and feeding,^31^ and the use of dirty clothes for wiping hands/utensils. A number of studies to date have assessed risk factors and microbial contamination in food in developing countries^12^,^32^–^35^; but few have developed or tested interventions to counter this problem in domestic settings.^36^,^37^

Changing people's behavior is a difficult and complex undertaking. Behaviors are determined by a wide array of factors; however, the few previous interventions that have been reported focused on imparting knowledge rather that changing behavior. Yet, work on other hygiene behaviors, such as handwashing, suggests that interventions using emotional drivers (such as nurture, disgust, affiliation, and status) may be more effective than those that teach about health benefits.^38^–^41^ We used Behavior Centered Design (BCD),^42^ a process of designing behavior change intervention underpinned by the Evo–Eco theory of change^43^ to design and evaluate a food hygiene behavior change intervention in rural Nepal. Though the country's health indicators are improving,^44^ diarrhea is still the second most important cause of death in under-fives and 41% of children are stunted.^44^

This study was designed to explore whether a systematically designed, scalable intervention underpinned by an explicit theory of change could improve multiple food hygiene behaviors in the challenging context of rural Nepal.

## Methods

### Study site.

Kavre District is located in the highland area of rural Nepal. Houses were made of mud and stone and the single ground floor room usually served for cooking, sitting, sleeping, and sometimes also for keeping animals. Most food preparation took place on the floor and firewood was the main source of fuel. Half of the population used water piped from unprotected springs, the other half surface water, and the majority of households had no toilet. Animal and child feces were visible throughout the study villages.

### Intervention design and delivery.

The food hygiene behavior change motivational package was designed following the five steps of BCD—A: Assess, B: Build, C: Create, D: Deliver, and E: Evaluate^42^.

Step A (assess): The first step involved the collection and analysis of published and local knowledge concerning food hygiene behavior to define target behaviors, the parameters of the intervention, and the questions to be answered in the Formative Research. We carried out a systematic review of literature on food hygiene (presented elsewhere), examined past experience, in particular small-scale weaning food studies in Mali,^37^ Brazil,^45^ and Bangladesh,^36^ other hygiene interventions, learning particularly from the World Health Organization five key behaviors for safer food^46^ and the successful SuperAmma handwashing trial in India.^38^ We consulted colleagues in government and non-governmental organizations to establish that the intervention would be replicable and scalable in the context of Nepal.

Step B (build): Formative research was conducted to investigate specific behaviors; target audiences, and behavioral determinants including habits, motives and plans, and social, physical, and biological factors in the kitchen and village environment (the key elements of the BCD model^42^,^43^). We carried out a Hazard Analysis and Critical Control Points assessment^47^ with microbial food testing to help us pinpoint behaviors that were a source of risk of food contamination. This work led us to identify the following five key behaviors to target:
  - Cleaning of child food-serving utensils using soap or ash before serving food
  - Handwashing with soap by mother before feeding, and by child before eating
  - Storing cooked food in containers with a tight-fitting lid
  - Thorough reheating of leftover/stored food before feeding to the child (temperature *P* ≥ 70°C)
  - Serving only treated water to the child.

**Figure 1.**
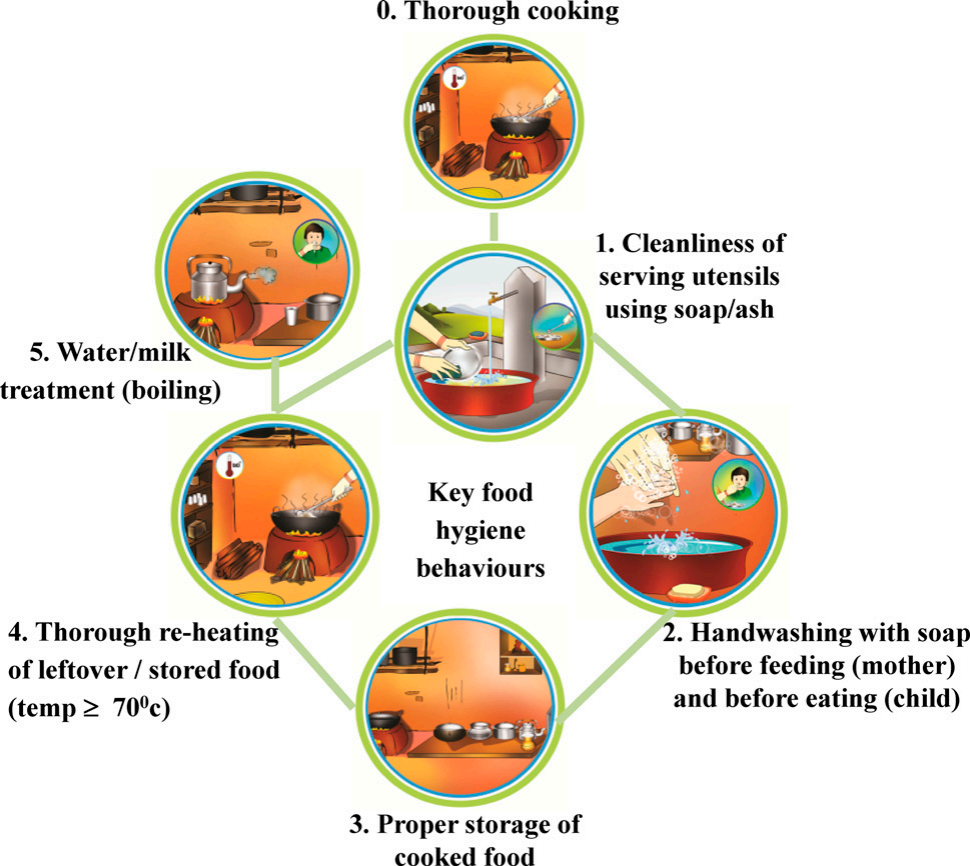
Five key prioritized food hygiene behaviors (from 1 to 5).

A further critical control point: the thorough cooking of foods to be fed to a child was also identified. However, this was already common practice, so did not need to be targeted. Figure 1

illustrates the five targeted behaviors.

Step C (create): A local creative team with expertise in marketing, design, innovation, program development, and behavior change was assembled to design the intervention. They were briefed to use the motives of nurture, disgust, affiliation, and status and to disrupt behavioral settings (social and physical determinants of behavior^43^,^48^). Key principles were that the intervention should be recognizable, feasible to implement by local health workers/volunteers, and have a reasonable possibility of replication at larger scale. Prototypes of the intervention components were developed and pretested in several iterations in nonstudy areas and the package was finalized after incorporating government and NGO stakeholder feedback.
Step D (deliver): The food hygiene promotion package was delivered through six events followed by six household visits from 15 trained Food Hygiene Motivators (FHMs) over a period of 3 months during May–August 2013 (see Table 1). The primary target audience were mothers with a child aged 6–59 months. The campaign's theory of change was that mothers would identify with a central “ideal mother” character, who practiced safe hygiene so as to be respected in the community (Status motive). Nurture, Disgust, and Affiliation were further levers of change, and we aimed to disrupt daily food preparation habits that were held in place by tradition, routine, and the social and physical settings of kitchens.

**Figure 2.**
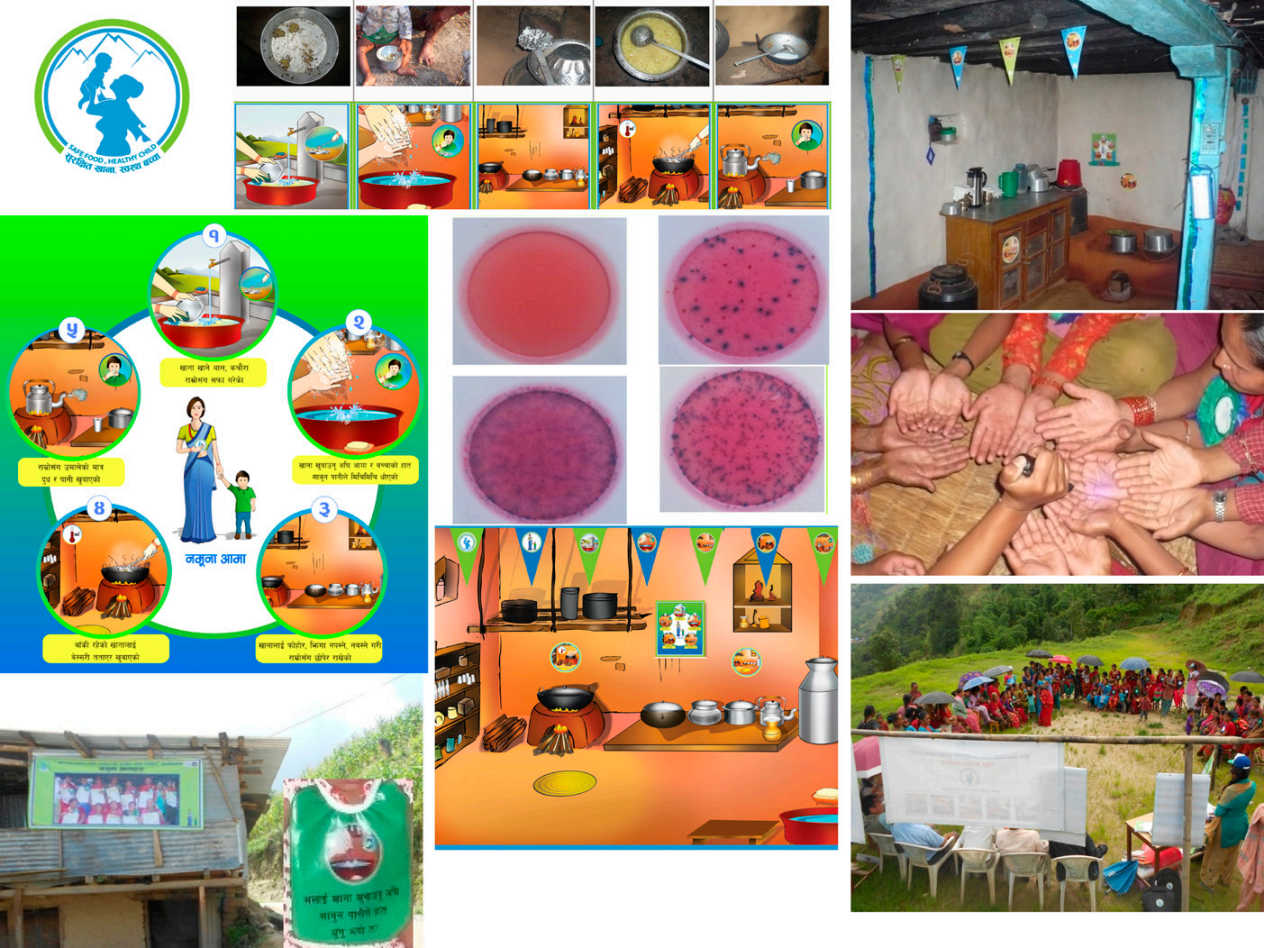
Images from the campaign materials and events.

FHMs with a similar profile to Nepal's ubiquitous Female Community Health Volunteers (FCHVs) were recruited locally and trained to implement the campaign. The details can be accessed online at http://www.shareresearch.org/om-prasad-gautam and a video documentary can be accessed at http://www.shareresearch.org/research/nepal-food-hygiene-intervention-campaign. Nurture-based activities included a game about the child's life, an exchange of letters, and a family drama. Affiliation-based activities included a folk song, a puzzle game, peer review, and cookery demonstration. Disgust-based activities included a Glo Germ demonstration and a hot potato game. Status-based activities included a public pledging, public display of photos of ideal mothers, and declarations of safe food zones. The social and physical settings of kitchens were disrupted by holding makeover parties where the kitchen was redecorated using colored bunting and danglers placed at eye level and when neighbors agreed to practice new hygiene rituals. Villages were then declared “safe food hygiene zone,” with volunteer mothers becoming food hygiene monitors. Figure 2

shows images from the campaign.

Step E (evaluate): Below we describe the evaluation of the intervention.

### Study population.

The evaluation was conducted between October 2012 and December 2013. Eight wards (clusters) were randomly selected from 18 eligible wards from two adjacent Village Development Committees of Kavre District. Study wards had to be rural, have a heterogeneous population, have to be geographically separated, have more than 30 households with a child aged 6–59 months, have low sanitation coverage, and have high diarrhea prevalence according to local health institution data. All households with at least one child aged 6–59 months in eight clusters became the study population.

### Recruitment, randomization, and masking.

Figure 3

**Figure 3.**
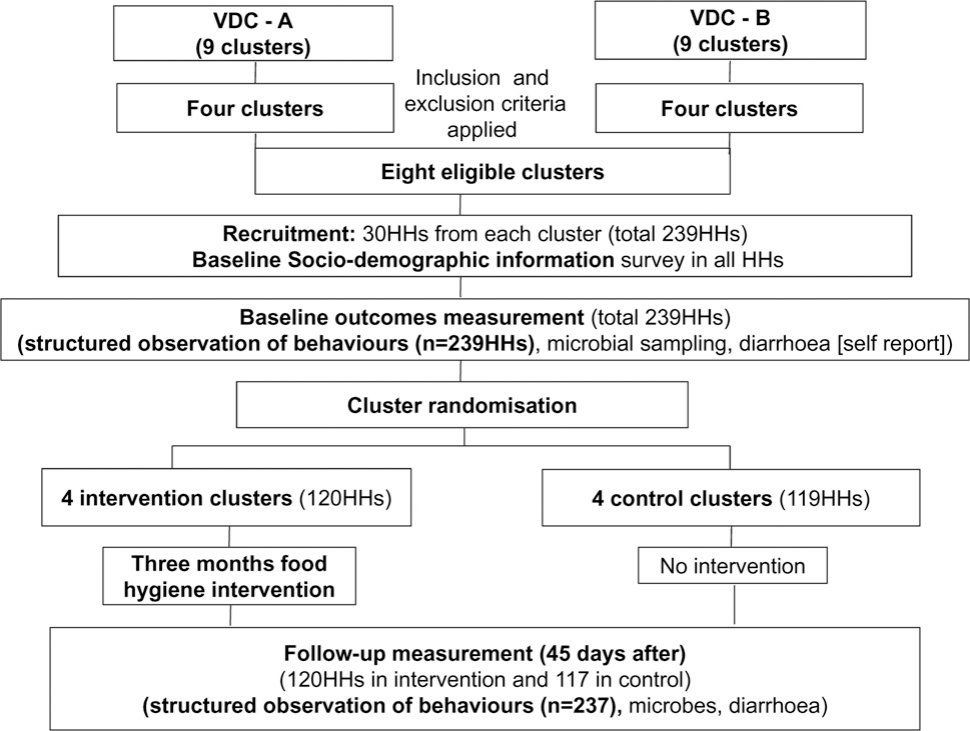
Flow diagram of the trial.

shows the flow diagram for the trial. Within each cluster, 29–30 households having at least one child aged between 6 and 59 months were randomly recruited to participate in the study. The final sample included 239 households from eight clusters. Written informed consent was received from all participating mothers. Social, demographic, and economic information was collected from each household using a closed-ended structured questionnaire.

The clusters were then randomized into four intervention and four control clusters. The intervention clusters received the food hygiene promotion intervention and no intervention was delivered in the control clusters. At baseline, there were 120 households with a child aged 6–59 months in the four intervention clusters, and 119 in the four control clusters. The primary outcome of interest (the proportion of mothers sustaining all key food hygiene behaviors) was measured by food hygiene observers not connected with the intervention. Mothers were told that the purpose of the observation was to document their daily routines.

### Outcome assessment.

The primary outcome—the proportion of mothers practicing all five target behaviors—was measured by structured observation.^38^,^40^ The proportion of mothers 1) cleaning child food serving utensils using soap/ash; 2) washing both hands with soap before feeding child and washing the child's hands before eating; 3) storing cooked food with tight lid and no visible flies/dust/dirt in the food; 4) thoroughly reheating leftover/stored food at adequate temperature (≥ 70°C); and 5) serving treated water to their children was assessed by direct observation (and temperature measurement). Assessments took place 45 days before and 45 days after completion of the 3-month intervention period. Twenty-five female food hygiene observers were recruited and trained to carry out the structured observation of food hygiene behaviors. Observations were made in all intervention households (*N* = 120, with no loss to follow-up) and control (nonintervention) households (*N* = 119 with two lost to follow-up) once at baseline and follow-up (postintervention). Observations were carried out between 1:00 pm and 5:00 pm, when the behaviors of interest were likely to be seen. Observations took place in both groups simultaneously, and were completed within 12 days. Observers were kept blind to the study objectives and had no role in the intervention. A structured observation checklist was used to record all behaviors. After the follow-up observation, the reach of the intervention was assessed to ascertain exposure density and to check for contamination of the control group.

### Sample size.

We calculated that a sample size of eight clusters with a minimum of 28 households per cluster for two sample comparisons of proportions using 95% confidence interval (*P* < 0.05), 90% power, 5% loss to follow-up (0.05), 1.29 design effect (due to village level clustering) would allow us to detect a difference of 20% (7% in control group, 27% in intervention group) in the prevalence of target behaviors between the control and intervention arms.

### Statistical analysis.

Our primary outcome was the comparison of the before/after change in cluster-level mean proportions of the observed practice of all five behaviors as a composite performance score between intervention and control clusters. We used cluster-level analysis since the intervention was allocated by cluster.^49^ As a secondary analysis, we compared all individual behaviors at cluster level by different groups during baseline and follow-up. Since we only had eight clusters, we used a nonparametric test, that is, the two-sample Wilcoxon rank-sum test (Mann–Whitney *U* test)^50^ to compare cluster-level means and to estimate statistical support. This test does not rely on the assumption of normality and is resistant to outliers.^49^ The effect size of the intervention was calculated by difference-of-differences, that is, [follow-up − baseline]_intervention_ minus [follow-up − baseline]_control_. Subgroup analysis stratified by religion, caste/ethnicity, educational level, economic status, and types of cooking fuel was carried out. Statistical support for effect modification was assessed by computing the difference in food hygiene behaviors (composite performance) between subgroups within each cluster, comparing the mean difference of differences between intervention and control clusters, following the method described by Cheung and others.^51^ The intra-class correlation coefficient was calculated using the STATA “loneway” command. Data were entered into a spreadsheet and SPSS, and statistical analysis was performed using SPSS 19 (IBM SPSS Statistics, Armonk, NY) and STATA 12 (StataCorp LP, College Station, TX).

### Ethics.

Ethical approval for the study was granted by the ethics committees of the London School of Hygiene and Tropical Medicine, United Kingdom, and the Nepal Health Research Council.

## Results

### Social and demographic characteristics.

Table 2 shows that intervention and control clusters and households had similar social and demographic characteristics. Clusters ranged in size from 75 to 141 households (417–786 people). The mean age of participating mothers was 27 years, and the majority lacked formal education. Over 50% of mothers in both groups belonged to the Hill Aadiwaasi/Janajaanati ethnic group (part of the historically deprived Tamang). Around one-third of mothers were of the Brahmin/Chhetri caste, and 8% were hill Dalit. Most households earned less than 10,000 Nepali Rupees per month (∼100 US$/month), mainly from agriculture. Only half of the participating households had latrines and around 65% of households reported disposing of their child's feces in fields. Animal feces were observed in 86% of household compounds. Soap was observed in more than 80% of households in both groups. Only one household had a refrigerator.

### Feeding practices.

The intervention and control group had similar feeding practices at baseline. Around 58% of children had received supplementary food before the age of 6 months. Children were fed with different types of liquid (water, raw cow, or buffalo milk), semi-solids (jaulo, lito-made from roasted rice flour, ghee, and sugar), solids (rice, dhido—a type of porridge with curry/pulse/vegetable), dry food (beaten rice, popcorn), and snacks (dry noodles). Some ethnic groups also fed jad (an alcoholic brew). The majority of households (86%) fed the same staple food to their children that they themselves consumed daily. Nine of 10 households cooked only twice a day, in the late morning and late evening, but children were generally fed four times a day with stored or leftover food. Food was cooked mostly by mothers and/or grandmothers and fed by hand to children.

### Reach of the intervention.

All mothers had heard of and participated in the campaign in the intervention group, compared with almost none in control cluster (see Table 3). Out of 12 expected exposures (two community events, four group events, and six household visits) during the 3-month campaign period, 90% of mothers were exposed at least 10 times. All intervention group mothers were able to describe the five key behaviors that “ideal mothers” should practice.

### Effects of the intervention on food hygiene behavior.

Figure 4

**Figure 4.**
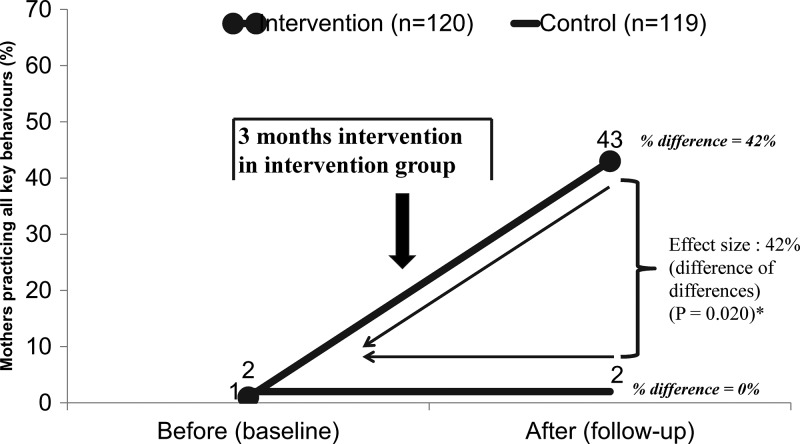
Key food hygiene behaviors before and after intervention, by study group. (Mean proportion of all key food hygiene behaviors [composite performance—cluster level analysis]: 1) serving utensils are washed using soap/ash just before putting child food, 2) mother washed her both hands with soap just before feeding child and child's both hands are washed before eating food, 3) stored all cooked/leftover food in container/s with a tight lid and no flies/no visible dirt–dust accessing stored food, 4) stored/leftover food are reheated before serving to child and maintained adequate temperature (70°C), and 5) served only treated water for their children when observed). * *P* value from Wilcoxon rank-sum test.

shows that the cluster average of mothers performing all five target behaviors was low in both intervention and control groups at baseline (1% [SD = 2%] versus 2% [SD = 2%]). Following the campaign, the key behaviors were more common in the intervention than in the control group (43% [SD = 14%] versus 2% [SD = 2%], *P* = 0.020; see Figure 4 and Table 4). After adjusting for the baseline prevalence, the effect size of the intervention (as a difference of differences) was an increase in the mean proportion of target behaviors of 42% (*P* = 0.020); see Table 4.

**Figure 5.**
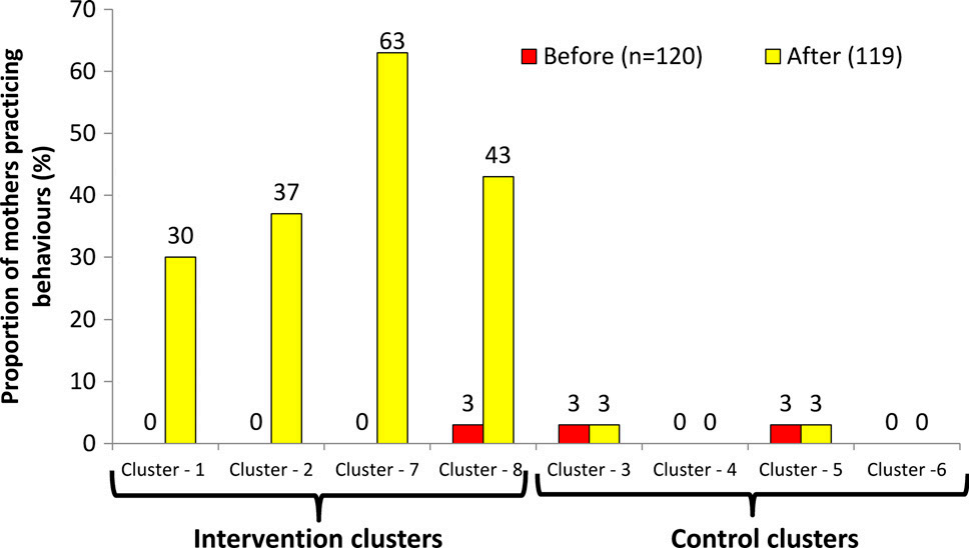
Key food hygiene behaviors before and after intervention, by study clusters. (Proportion of mothers sustaining all key food hygiene behaviors by cluster: 1) serving utensils are washed using soap/ash just before putting child food, 2) mother washed her both hands with soap just before feeding child and child's both hands are washed before eating food, 3) stored all cooked/leftover food in container/s with a tight lid and no flies/no visible dirt-dust accessing stored food, 4) stored/leftover food are reheated before serving to child and maintained adequate temperature (70°C), and 5) served only treated water for their children when observed).

Target behaviors improved in all intervention clusters from 0% to 30% (*P* = 0.002) in cluster 1, from 0% to 37% (*P* = 0.001) in cluster 2, from 0% to 63% (*P* < 0.001) in cluster 7, and from 3% to 43% (*P* = 0.001) in cluster 8 (Figure 5

). There was no difference between baseline and follow up among the control clusters.

Figure 6

**Figure 6.**
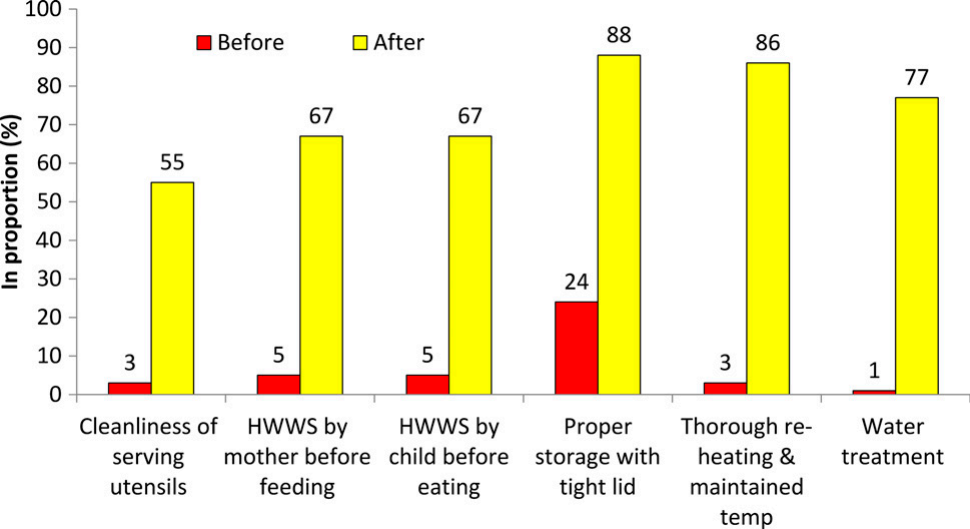
Prevalence of key food hygiene behaviors before and after the food hygiene campaign in intervention group (*N* = 120).

and Table 4 gives the changes in each targeted behavior. The proportions of mothers practicing each behavior in intervention and in control groups at baseline were similar. All behaviors improved in the intervention groups, but not in the control groups. The intervention increased the mean proportion correctly reheating food by 85% (*P* = 0.020). The mean temperature of reheated food was 54°C (minimum 30°C–maximum 75°C) in the intervention and 61°C (minimum 35°C–maximum 78°C) in the control group at baseline. Following the intervention, the mean temperature was 76°C (minimum 55°C–maximum 92°C). The effect size of the intervention (as difference of differences) for each of the targeted behaviors are shown in Table 4. Though there appeared to be some difference in effect size for the combined behavior score with education, ethnicity, and type of cooking fuel, statistical tests showed little statistical support.

The intra-class correlation coefficient of key food hygiene behaviors (effect of all behaviors) at village level was 0.000 at baseline and 0.043 at follow-up. At household level, the intra-class correlation coefficient was 0.000 at baseline and 0.475 at follow-up.

## Discussion

This study suggests that it is possible to change entrenched food hygiene habits, even in environmentally challenging conditions such as pertain in rural Nepal. We attribute the apparent success of the intervention to the use of a systematic process employing global and local knowledge, behavioral theory, and a creative process to design a tailored intervention targeting emotional drivers of food hygiene behavior as well as disrupting food preparation settings.

An alternative interpretation of the study results is that the changes in behavior that were observed were not representative of real behavior, but were due to reactivity on the part of observed mothers. In effect, mothers may have been anxious to demonstrate to observers that they had learnt the lessons of the campaign by adhering closely to target behaviors during the observation period, but did not actually change their daily food hygiene routines. We think that this is unlikely because 1) observers were not connected by mothers to the intervention and mothers were told that the observation was to monitor their daily routine and 2) the analysis of microbiological samples taken at unannounced visits support the findings of improved behaviors (results presented elsewhere). Similar studies on the effectiveness of handwashing with soap interventions in Bangladesh^41^ and India^38^,^40^ also used direct observation. Those studies claimed that the differential reactivity was likely to be low if participants saw no link between the intervention and the outcome measurement process.

The fact that the intervention was equally effective across targeted behaviors and across clusters and in differing socioeconomic settings suggests that the improvements were due to the effects of the intervention itself. The intervention was based on motivating mothers rather than educating them using a creative approach and behavior change science. Several authors have called for the use of more creative and innovative techniques to change public health-related behavior.^38^,^40^,^41^,^52^,^53^ We also paid attention to the training and motivation of our outreach workers (FHMs) as studies have suggested that the quality of interventions improves if the implementation team is skilled.^38^ Several of our FHMs were also FCHVs. They found that it was feasible to deliver the intervention on top of their existing workload, suggesting that the package might capable of being scaled across Nepal.

The question of which elements of the intervention were most effective cannot be determined from this quantitative study. Hence, we do not know if the kitchen makeovers or the activities based on nurture, disgust, affiliation, or status were the most active ingredients of the intervention. We suspect that the food preparation setting disruption activity was particularly effective, involving as it did a transformation of the physical environment (repainting, bunting, danglers as behavior reminder, kitchen tools), the script (mothers committing to behave in a new way), social control (commitment made in front of their neighbors), and the changing designation/purpose of the setting (from kitchen corner to safe food hygiene zone). Figure 2 (right top picture) shows a typical transformation from all-purpose room used for cooking, eating, sleeping, and keeping animals to a beautiful, bright, and special small kitchen in one corner. The settings idea is a powerful one that was laid out in the 1950s by ecological psychologists,^48^ but that has since been neglected. Yet Roger Barker showed that knowing settings can predict behavior 90% of the time, and all behavior takes place in settings.^54^ This concept could be useful for changing health-related behavior.

The use of emotional motivators such as nurture, disgust, affiliation, and social status may have motivated the key behaviors. Our focus on the positive emotional reward of each behavior (becoming an ideal mother, shiny serving utensils, child's warm tummy, tasty food, social approval) probably helped to reinforce each behavior, making them part of the daily food preparation routine. To our knowledge, this is the first study to attempt to use emotional drivers to affect food hygiene behaviors, though they have been shown to work on other hygiene behaviors such as handwashing in low-38,39,55 and high-income^56^,^57^ countries.

This study faced the particular challenge of trying to change five different behaviors at the same time. Our intervention was designed to disrupt their common setting and used drivers that could be associated with all five behaviors. We further suspect that performing one behavior served as a reminder to perform another. For example, mothers practicing cleanliness of serving utensils just before feeding their children were likely to remember to wash their hands just before feeding, as both activities happened simultaneously. Many of the target behaviors happened in sequence; for example, immediately after reheating the food, the mother served the food using serving utensils, then washed her hands, and stored the leftover food properly. It may thus be easier to change multiple behaviors when they are practiced in similar settings and in sequence, when the practice of one can cue another.

The intervention was relatively intense, with six events and six door-to-door contacts. Based on our process evaluation (forthcoming), the intervention could be simplified for wider scaling-up at reduced cost. This study, however, provides proof of principle that food hygiene can be improved in challenging environments provided that interventions are based on a careful process involving Formative research, behavioral theory, and imaginative and motivating creative campaigns. BCD provides a simple process framework for the design of such interventions (the ABCDE steps) as well as theoretical basis for identifying key drivers of and a theory of change for behavior. The intervention was relatively intense, however, and it remains to be seen if the large-scale replication of the package will achieve the same degree of behavior change and whether such changes in behavior can be sustained for the long term.

## Figures and Tables

**Table 1:** Summary of the intervention components

Events/visits	Purpose	Key content
First community event (3 hours)	Raise awareness of, generate interest in, and elicit commitment to the campaign and the five food hygiene behaviors	Distributed invitation card a day before the event. Program ritual (put-up back-drop banner, nail cutting, hand washing with soap, putting on program badge, etc.) initiated. Program jingle introduced. Campaign objectives described by social leader. Situation contextualized via situational analysis—story, flex with pictures, video clip (disgust motive exploited). Five food hygiene behaviors and their benefits presented. “Ideal mother” introduced as a source of inspiration. Public commitment oath taken, and certificates distributed. Public rally chanting—“safe food, healthy child, we want ideal mother”
First households visits (3 hours)	Remind mothers of public commitment; change settings reinforcing the desired behaviors (particularly kitchen cleanliness)	Program jingle installed on mothers' phones. Kitchen compared with “ideal kitchen”—using clean kitchen illustration. Kitchen demarcated with ribbons and flags reminding mothers of the food hygiene behaviors. Danglers placed at eye-level (round illustration of all behaviors, ideal mother board, *dhungro*—a branded fire blowing instrument). Importance of food hygiene behaviors refreshed via a brief talk using a three-dimensional flip chart. Three-month work plan formulated to ensure each mother meets the public commitment
First group event (3 hours)	Reinforce program ritual; establish group norms/habits for all behaviors; and generate interest in having clean kitchens	Program ritual carried out. Mothers' experiences of changes in their kitchens shared. Group norms elicited via cooking demonstration. Benefits of five food hygiene behaviors reiterated via visual aids (3M PetriFilm [3M, St. Paul, MN], Glo Germ lotion [Hygienic Solutions, Lincoln, United Kingdom] before feeding). Bibs with the message “did you wash your hands before feeding me?” distributed as reminder/reward for HWWS. “Clean kitchen” competition announced (putting clean kitchen indicators in the village)
Second household visits (2 hours)	Reinforce correct food hygiene behaviors with the view to these becoming habitual	Mothers' preparation of food observed and corrected where necessary. Importance of five food hygiene behaviors reiterated (used 3M PetriFilm, Glo Germs, bib, plastic bucket for handwashing, kettle for boiling water). Mothers reminded about “clean kitchen” competition
Second group event (3 hours, 15 minutes)	Increase mothers' confidence; link food hygiene behaviors with affiliation, nurture, and status; generate interest in becoming an “ideal mother”	Program ritual carried out. Obstacles faced by mothers shared and strategies for overcoming these discussed. “Child Life Game” played—the future that mothers want for their children discussed and linked to the five food hygiene behaviors (nurture motive). Puzzle game played to encourage kitchen cues (social respect motive). Folk song composed by mothers conveying key food hygiene messages—affiliation elicited. “Ideal mother” competition announced. Behavior reminder “fan” reflecting five behaviors and ideal mother sticker distributed
Third household visits (2 hours)	Establish reheating and boiling as social norms; ensure a conducive family environment exists to practiced behaviors	Mothers' food reheating practices observed and corrected where necessary (noting reheated temperature, motivated to use appropriate vessel to reheat food, and kettle to boil water). Family meeting held to promote food hygiene behaviors (using 3D flip chart). Mothers reminded about “clean kitchen,” and “ideal mother” competitions (visual cues). Unidentical visits performed (by field staff and coordination committee)
Third group event (3 hours)	Show that implementing the five food hygiene behaviors will avoid disgust and social exclusion and will increase social prestige and happiness	Program ritual carried out. Mothers participated in disgust exercises (Glo Germs used in food, plate, bowl, glass, spoon) and games (hot potato game using disgusting and safe pictures to demonstrate social inclusion and exclusion). “Safe food hygiene zone” competition announced. “Clean kitchen” competition winner announced and publically commended, thereby conferring = prestige. Participants and guests visited winner's house to encourage and share learning
Fourth household visits (2 hours)	Create peer pressure, build confidence, and reduce observer bias in observation of mothers' five behaviors	Peer-review (watch-dog) exercise carried out (element of secrecy entailed) by peer mother. Observer mother reported back practices. Mothers reminded about “ideal mother” and “safe food hygiene zone” competitions. Mothers' three-month work plans reviewed. Unidentical visits performed (by field staffs and coordination committee)
Fourth group event (2 hours, 30 minutes)	Reiterate that implementing the five food hygiene behaviors will increase social prestige and status; encourage men to participate	Program ritual carried out. Advice provided by mothers to a fictional mother (Dhukhimaya) experiencing social, environmental, and attitudinal barriers to adopting food hygiene behaviors. A drama (family member role play) showed how to become an ideal mother and tacking social, attitudinal, and physical barrier. “Ideal mother” competition winners announced and publically commended (ideal mother photo placed in the junction of the village), thereby conferring prestige. Men involved in the event and celebration
Fifth household visits (1 hour)	Reinforce food hygiene behaviors; mothers self-evaluate their food hygiene behaviors	Mothers' work plans reviewed. Mothers' food hygiene behaviors observed (ongoing progress). Mothers' performance self-evaluated publically. “Safe food hygiene zone” indicators reinforced
Second community event (4 hours)	Ensure food hygiene behavior change is sustainable postintervention by further entrenching them as social norms and prestige-conferring practices	Program ritual carried out. Response received from Dhukhimaya linking food hygiene behaviors to child health and social status. Encouraging social norms by reperforming folk song, etc. Mothers volunteer to continually monitor community's food hygiene behaviors. Mothers publically repledge their commitment to sustainable food hygiene behavior change (appreciation certificate distributed). Experiences of stakeholders heard. Remarks from social leaders, guests, representatives link food hygiene as to social respect. “Safe food hygiene zones” declared and bill boards erected at each entry point of the cluster. Group photo session performed. Community rally chanting “we want ideal mother, ideal mother hi-hi, diarrhea bye-bye) and using local music and program song. Intervention formally closed
Sixth household visits (1 hour)	Entrench food hygiene behaviors into mothers' daily routines and identify any remaining barriers to these practices; ensure sustainability	Sustainability work plans formulated by mothers. Ease of implementation of food hygiene behaviors analyzed by participants (pile shorting exercise using illustrations) and feedback provided. Sustained behavior change pledged by entire families. Household visits formally end

For more details about program activities components, follow blog: http://www.shareresearch.org/om-prasad-gautam.

**Table 2:** Social and demographic characteristics of the study population at baseline

Variable	Control (*N* = 119 HH)	Intervention (*N* = 120 HH)	*P* value*
Village/cluster size (mean, range)	95 (83–112)	99 (75–141)	
Number of clusters	4 (100%)	4 (100%)	
Selected HHs per cluster (mean, range)	30 (29–30)	30 (30–30)	
Family size (mean, SD)	5.8 (2.3)	5.9 (2.1)	
Mothers' age (mean, range)	27 (18–50)	27 (19–43)	
Number of children (6–59 months)	143 (100%)	150 (100%)	
Religion (%)			
Hinduism	48 (40)	59 (49)	0.553
Buddhism	71 (60)	59 (49)	
Others	0 (0)	2 (2)	
Education level of mothers (%)			
None or informal	62 (52)	58 (48)	0.816
Primary (up to 5th grade)	23 (19)	27 (23)	
Secondary (up to 10th grade)	26 (22)	24 (20)	
Higher secondary or university	8 (7)	11 (9)	
Caste/ethnicity of mothers (%)			
Brahmin/Chhetri/Thakuri	34 (29)	46 (38)	0.743
Hill Aadiwaasi/Janajaati	76 (64	64 (53)	
Hill Dalit	9 (8)	10 (8)	
Monthly HHs income in NRs (%)			
< 10,000 NRs	70 (59)	63 (52)	0.622
≥ 10,000 to < 20,000 NRs	30 (25)	37 (31)	
≥ 20,000 NRs	19 (16)	20 (17)	
Types of cooking fuel (%)			
Firewood	111 (93)	104 (87)	0.421
Gas cylinder	3 (3)	3 (3)	
Bio-gas	5 (4)	13 (11)	
Main water source for drinking (%)			
Piped water to tap in yard, plot	57 (48	62 (52)	0.810
Surface water	62 (52)	58 (48)	
Toilet/latrine at households	64 (54)	60 (50)	0.703
Soap observed at HHs	100 (84)	96 (80)	0.519
Refrigerator at households	1 (1)	1 (1)	0.995

NR = Nepali Rupees; SD = standard deviation.

* *P* value from χ^2^ test after clustering (cluster level analysis).

**Table 3:** Reach of the intervention—postintervention measurement

Variable	Control (*N* = 117 HH) (%)	Intervention (*N* = 120 HH) (%)
Heard of food hygiene intervention?	1	100
Participated in food hygiene campaign?	0	100
Participated in > 10 events/HH visits (*N* = 12)	0	90
Exposure by intervention cluster in > 10 events/HH visits		
Cluster 1	–	97
Cluster 2	–	90
Cluster 7	–	90
Cluster 8	–	83
Participated in competitions?		
Clean kitchen competition	0	100
Ideal mother competition	0	100
Safe food hygiene zone	0	99
Made public commitment to practice behaviors?	0	95
Made public commitment to sustain behaviors?	0	93
Reported that the ideal mother should practice following behaviors?		
Cleanliness of serving utensils	1	100
HWWS before feeding and eating	1	100
Proper storage of leftover food	1	100
Thoroughly reheat leftover/stored food	1	100
Treat water and boil milk before serving	1	100
Reported belief that social norms changed over time in village as the following became more common		
Cleaning serving utensils just before feeding?	2	91
HWWS before feeding child	8	97
Storing food in container with a lid	21	98
Reheating food before eating	9	98
Boiling/treating water before drinking	3	85

**Table 4:** Changes in mother's food hygiene behaviors from baseline to follow-up period (direct comparison and difference of differences)

S. No.	Key food hygiene behaviors	Baseline*			Follow-up*			Effect size (difference of differences)			
		Intervention (*N* = 120)	Control (*N* = 119)	*P* value†	Intervention (*N* = 120)	Control (*N* = 117)	*P* value†	% difference: intervention	% difference: control	% difference of differences (%)	*P* value†
Composite performance (behavior)											
All	Proportion (in mean) of mothers sustaining all key food hygiene behaviors (combination of all key behaviors)	1% (2 SD)	2% (2 SD)	0.4047	43% (14 SD)	2% (2 SD)	0.020	42	0	42	0.020
Individual behaviors (five key behaviors)											
1	Proportion of mothers cleaning child food serving utensils using soap/ash just before putting child's food	3% (4 SD)	6% (6 SD)	0.544	55% (16 SD)	4% (4 SD)	0.019	52	−2	54	0.021
2a	Proportion of mothers washing both hands with soap and water before feeding child	5% (4 SD)	7% (3 SD)	0.457	67% (15 SD)	5% (7 SD)	0.020	62	−2	64	0.021
2b	Proportion of child washed both hands with soap and water before eating food	5% (4 SD)	5% (4 SD)	0.883	67% (17 SD)	4% (5 SD)	0.021	62	−1	63	0.021
3	Proportion of households stored cooked/leftover food in containers with a tight-fitting lid and no flies/no visible dirt–dust in stored food	24% (17 SD)	26% (16 SD)	0.885	88% (11 SD)	21% (15 SD)	0.020	64	−5	69	0.021
4	Proportion of mothers thoroughly reheating leftover/stored food and maintaining 70°C or > 70°C	3% (4 SD)	6% (3 SD)	0.240	86% (8 SD)	4% (2 SD)	0.019	83	−2	85	0.020
5	Proportion of households treating water before serving to child	1% (2 SD)	3% (2 SD)	0.155	77% (6 SD)	5% (5 SD)	0.021	76	2	74	0.021

SD = standard deviation. Data collapsed to analyze at cluster level.

* Mean proportion.

† Wilcoxon rank-sum test (Mann–Whitney).
